# Supplementary material for: The interactome of intact mitochondria by cross-linking mass spectrometry provides evidence for coexisting respiratory supercomplexes
Source: Mol Cell Proteomics. 2017 Dec 8;17(2):216–32. doi: 10.1074/mcp.RA117.000470 (PMC5795388; doi:10.1074/mcp.RA117.000470)
Supplement: Supplemental Data [file supp_RA117.000470_133922_0_supp_23978_fzffwf.zip › spectra_annotation/mito_spectra_annotation/477-1-10-1-8-1.pdf]

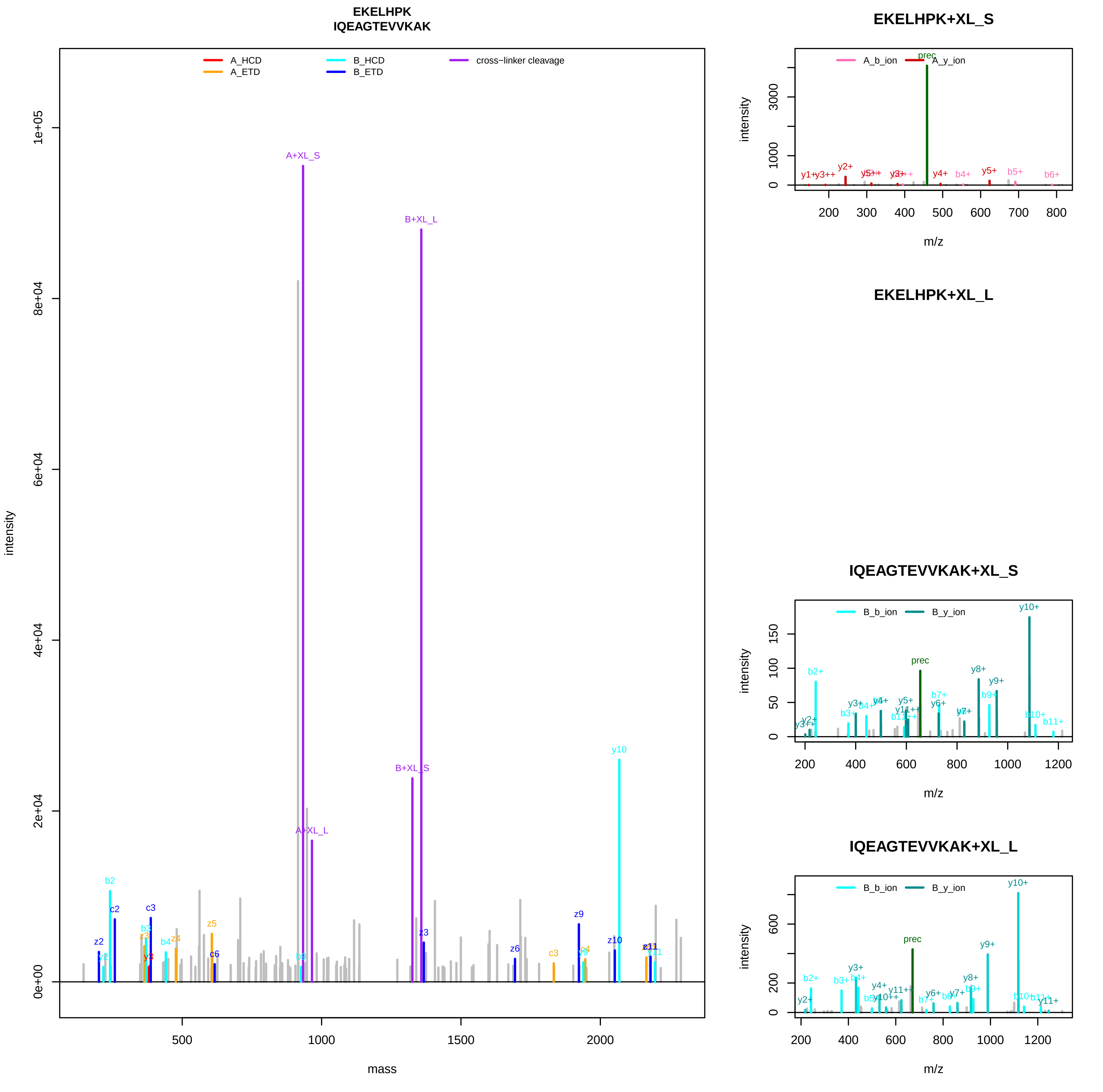
EKELHPK
IQEAGTEVVKAK
A_HCD
A_ETD
B_HCD
B_ETD
cross−linker cleavage
A+XL_S
B+XL_L
B+XL_S
A+XL_L
y10
b2
c2
c3
z2
z9
z10
z6
z3
z5
c3
c6
b4
z4
intensity
mass
0e+00
2e+04
4e+04
6e+04
8e+04
1e+05
500
1000
1500
2000
EKELHPK+XL_S
A_b_ion
A_y_ion
prec
y2+
y4+
y5+
b4+
b5+
b6+
m/z
EKELHPK+XL_L
IQEAGTEVVKAK+XL_S
B_b_ion
B_y_ion
y10+
prec
b2+
y8+
y9+
b9+
b10+
b11+
IQEAGTEVVKAK+XL_L
y10+
y9+
prec
b2+
y8+
y3+
b3+
b4+
